# Ideal Cardiovascular Health Metrics Are Associated with Reduced Severity of Hepatic Steatosis and Liver Fibrosis Detected by Transient Elastography

**DOI:** 10.3390/nu14245344

**Published:** 2022-12-16

**Authors:** Heze Fan, Chenbo Xu, Wenyuan Li, Yuzhi Huang, Rui Hua, Ying Xiong, Yuxuan Yang, Xueying Feng, Zihao Wang, Zuyi Yuan, Juan Zhou

**Affiliations:** 1 Cardiovascular Department, First Affiliated Hospital of Xi’an Jiao Tong University, Xi’an 710061, China; 2 Key Laboratory of Environment and Genes Related to Diseases, Ministry of Education, Xi’an 710061, China

**Keywords:** National Health and Nutrition Examination Survey, cardiovascular health metrics, liver stiffness measurement, controlled attenuation parameter, healthy lifestyle

## Abstract

Life’s Simple 7 (LS7) is the American Heart Association’s (AHA) proposal for a healthy lifestyle, also known as cardiovascular health (CVH) metrics. However, the association between CVH metrics and the severity of hepatic steatosis and liver fibrosis detected by transient elastography is unknown. We performed a cross-sectional study using the data from the 2017–2018 National Health and Nutrition Examination Survey (NHANES) cycle. The controlled attenuation parameter (CAP) and liver stiffness measurement (LSM) were used to evaluate the severity of hepatic steatosis and liver fibrosis and to define NAFLD, advanced liver fibrosis, and cirrhosis. A total of 2679 participants were included. Multivariate linear regression analysis revealed that per 1-unit increase in the CVH metric, CAP and LSM decreased by 8.565 units and 0.274 units, respectively. In the multivariate logistic regression analysis, the risk of NAFLD, advanced liver fibrosis, and cirrhosis were 7, 10, and 6 times higher in the poor CVH group than in the ideal CVH group. Subgroup analysis indicated that CVD patients and non-Hispanic whites could benefit more from ideal CVH. In conclusion, adherence to ideal CVH metrics, as proposed by the AHA, can significantly reduce the risk of hepatic steatosis and liver fibrosis.

## 1. Introduction

Non-alcoholic fatty liver disease (NAFLD) is a group of liver diseases caused by metabolic abnormalities characterized by liver fat accumulation, ranging from hepatic steatosis to non-alcoholic steatohepatitis (NASH), which can eventually progress to cirrhosis [1]. A meta-analysis revealed that nearly a quarter of the world’s population had NAFLD, which has become a significant public health problem [2]. Given that there are no specific drugs for NAFLD, lifestyle changes remain the cornerstone to inhibit the progression of NAFLD. In 2010, the American Heart Association (AHA) published guidelines called Life’s Simple 7 (LS7) or cardiovascular health (CVH) metrics to reduce the burden of cardiovascular disease (CVD) [3]. LS7 defines four health behaviors and three health factors, advocating that a healthy lifestyle should include avoiding smoking, regular physical activity, maintaining a normal weight, healthy eating patterns, and reasonably controlling blood pressure, blood sugar, and blood lipids. Emerging studies have reported that participants with ideal CVH metrics tended to have a lower risk of NAFLD [4,5,6,7,8]. However, since these studies used abdominal ultrasound, CT scans, or fatty liver index (FLI) calculated based on triglycerides and gamma-glutamyl transferase (GGT) to diagnose NAFLD, the severity of hepatic steatosis could not be assessed. Furthermore, few studies have explored the link between liver stiffness and ideal CVH metrics. Only two studies from Asia examined the relationship between CVH metrics and liver fibrosis, which is defined based on a non-alcoholic fatty liver fibrosis score (NFS) calculated using biomarkers (AST, ALT, albumin, and platelet count), age, BMI, and diabetes [6,7]. Similar to FLI, NFS also generally only determines the presence of advanced liver fibrosis and does not reflect the severity of the liver disease because it is not a direct measure of the liver. Therefore, the association between the severity of hepatic steatosis and liver fibrosis and the CVH metrics remains unclear.

Vibration-controlled transient elastography (VCTE) measures the velocity of the mechanically generated shear wave through the liver, resulting in a liver stiffness measurement (LSM), a marker of liver fibrosis [9]. While measuring the LSM, the controlled attenuation parameter (CAP), a marker of hepatic steatosis, was obtained by measuring the attenuation of the ultrasound signal through the liver [10]. Some articles have demonstrated that the VCTE had better diagnostic accuracy compared to scoring systems derived from liver biomarkers [11,12]. Since in the 2017–2018 cycle of the National Health and Nutritional Examination Survey (NHANES), VCTE was conducted for the first time to assess liver health among US adults, we wanted to use the data from the cycle to explore the role of CVH metrics, representing the combined effect of multiple lifestyle modifications, on the severity of hepatic steatosis and liver fibrosis detected by VCTE.

## 2. Materials and Methods

### 2.1. Study Population

The study included individuals from the NHANES during 2017–2018, a nationwide cross-sectional survey conducted by the Center for Disease Control and Prevention (CDC) to evaluate the health status of Americans. Of the 9254 participants included in the NHANES (2017–2018), we excluded the following participants: (1) participants with missing CAP or LSM data (*n* = 3306), (2) participants with missing CVH metrics’ component data (*n* = 2661), (3) participants with hepatitis B or C (*n* = 77), (4) participants with significant alcohol intake (≥4 drinks/d for females and ≥5 drinks/d for males) (*n* = 338), (5) participants who lacked information on covariates (*n* = 193). Finally, our study enrolled 2679 participants.

### 2.2. CVH Metrics

CVH was assessed based on the following 7 behaviors and risk factors: smoking status, body mass index (BMI), physical activity, healthy diet score (HEI), total cholesterol, blood pressure, and fasting plasma glucose (FPG). Each metric was categorized as poor, intermediate, or ideal and assigned scores of 0, 1, and 2, respectively [3]. The overall CVH score was the sum of the scores for the 7 metrics (range 0 to 14). The total CVH score was also divided into poor (0–7), intermediate (8–10), and ideal (11–14) [3].

Smoking status was categorized as ideal (never smokers: less than 100 cigarettes in life), intermediate (former smokers: more than 100 cigarettes in life and smoke not at all now), and poor (currents smokers: smoked more than 100 cigarettes in life and smoked some days or every day). Physical activity was categorized as ideal (≥8000 metabolic equivalent of task (MET) min/week), intermediate (600–7999 MET min/week), and poor (<600 MET min/week) [13]. Diet quality was assessed using the latest iteration of the HEI (2015), which consists of 13 components [14]. For the nine recommended components (total fruits, whole fruits, total vegetables, greens and beans, whole grains, dairy, total protein foods, seafood and plant proteins, and fatty acids), higher scores are associated with higher consumption levels. For the next four components that should be consumed sparingly (refined grains, sodium, added sugars, and saturated fats), higher scores are associated with lower consumption levels. HEI-2015 scores are not calculated based on absolute amounts of components but on the energy density per 1000 kcal (except for fatty acids). The fatty acid score was obtained from the unsaturated to saturated fatty acids ratio. The total HEI-2015 scores range from 0 to 100. Diet quality was classified as ideal (HEI-2015 ≥ 81), intermediate (50–81), and poor (HEI-2015 < 50) [15].

For total cholesterol, ideal, intermediate, and poor health was defined as <200, 200 to 239, and >239 mg/dL, respectively. Total cholesterol was measured using a Roche Modular P chemistry analyzer. We used hemoglobin A1c (HbA1c) to classify participants due to the high percentage of participants lacking FPG data, consistent with previous studies. We defined ideal, intermediate, and poor health by HbA1c values < 5.7%, 5.7%–6.4%, and >6.4%, respectively [15,16]. HbA1c was measured using a Tosoh G8 Glycohemoglobin Analyzer. For blood pressure, according to current clinical practice guidelines, average systolic (ASBP) and diastolic blood pressures (ADBP) were used to categorize participants into ideal (ASBP < 120 mmHg and ADBP < 80 mmHg), intermediate (ASBP 120–129 mmHg and ADBP < 80 mmHg), and poor (ASBP ≥ 130 mmHg or ADBP ≥ 80 mmHg) [17]. If participants reported the use of lipid-lowering medications, antihyperglycemic medications, or antihypertension medications, they were classified as intermediate (if they met ideal criteria) or poor (if they did not meet ideal criteria). Participants with BMI < 25 kg/m^2^, 25 to <30 kg/m^2^, and ≥30 kg/m^2^ were considered as having ideal, intermediate, and poor health status, respectively.

### 2.3. Definition of Advanced Liver Fibrosis, Cirrhosis, and NAFLD

The FibroScan^®^ model 502 V2 Touch equipped with a medium (M) or extra-large (XL) wand (probe) was used to obtain the elastography measurements at the NHANES Mobile Examination Center (MEC). With FibroScan^®^, a gentle, low frequency (50 Hz) mechanical vibration was transmitted to the intercostal area through a vibrating tip that touches the skin, which induced a shear wave through the liver. The velocity of the shear wave was converted into liver stiffness. Furthermore, the ultrasound attenuation signal, related to hepatic steatosis, was also measured by FibroScan. The exam was regarded as reliable if participants fasted at least 3 h prior to the exam and obtained more than 10 LSM values with an interquartile range/median < 30%. According to previous studies, advanced liver fibrosis (≥F3) and cirrhosis (F4) were defined as a median LSM (liver stiffness measurement) ≥ 9.7 kPa and ≥13.6 kPa, respectively [18]. Furthermore, we defined NAFLD as a CAP score ≥ 285 dB/m [19].

### 2.4. Covariates

Sociodemographic characteristics were collected using the sample person and family demographics questionnaires. Participants were considered to have a history of CVD if they reported coronary artery disease, angina pectoris, congestive heart failure, stroke, or heart attack. BMI was obtained by dividing the weight (kg) by the square of height (m^2^). We used the Chronic Kidney Disease Epidemiology Collaboration (CKD-EPI) formula to compute the estimated glomerular filtration rate (eGFR) [20]. The DcX800 method was used to measure liver biomarkers, including aspartate aminotransferase (AST), alanine aminotransferase (ALT), albumin, and total bilirubin concentration. Platelet count was measured using the Beckman UniCel DxC800 Synchron. The Roche Modular P chemistry analyzer and the Roche Cobas 6000 were used to measure high-density cholesterol (HDL) and high-sensitivity C-reactive protein (Hs-CRP), respectively. Detailed information on the laboratory methods can be found at: https://wwwn.cdc.gov/Nchs/Nhanes/2017-2018/BIOPRO_H.htm (accessed on 1 October 2022).

### 2.5. Statistical Methods

Variables are presented as mean with standard deviations (continuous variables) or percentages (categorical variables) as appropriate. Descriptive statistics were presented according to the categories of CVH metrics, and subgroup differences were explored by chi-square, ANOVA, or Kruskal–Wallis H-test as appropriate. We performed the linear regression model to explore the association between CAP, LSM, and CVH metrics. Furthermore, the logistic regression model was also performed when considering the outcome variables were NAFLD, advanced liver fibrosis, and cirrhosis. We treated CVH metrics as both continuous and categorical variables in the regression model. We performed three regression models: model 1: unadjusted model; model 2: age, gender, and race adjusted; model 3: adjusted for all covariates. We also included all components of the CVH metric in model 3 to evaluate the relationship between CAP, LSM, and each component. Furthermore, the restricted cubic spline was performed to visualize the association between NAFLD, advanced liver fibrosis, cirrhosis, and CVH metrics. To explore whether the association was modified by age (<60 and ≥60 years), sex, race, and CVD status, we also performed stratified analyses. A *p*-value < 0.05 was considered statistically significant. R (version 4.1.0, Vienna, Austria) software was used to perform all statistical analyses.

## 3. Results

### 3.1. Baseline Characteristic

**Figure 1:**
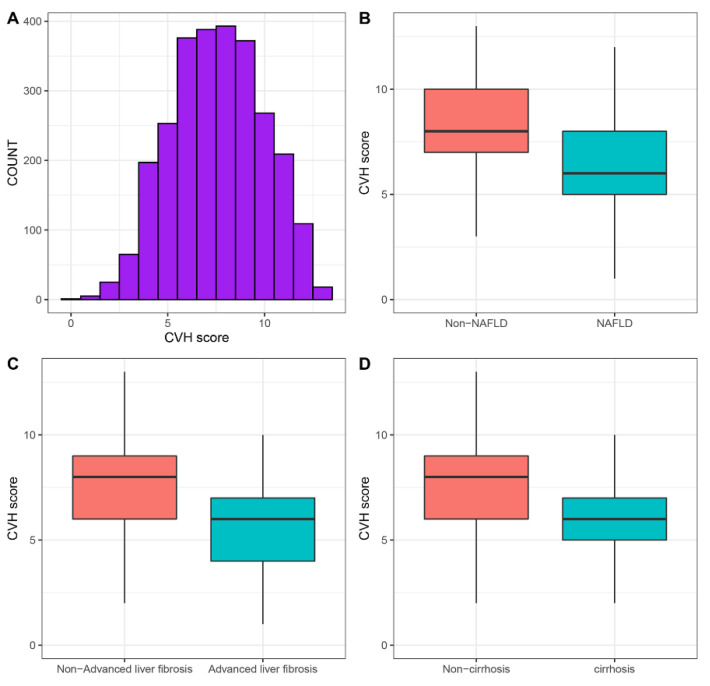
The distribution of CVH metrics. (**A**) The distribution of CVH metrics in the total population. (**B**) CVH metrics in participants with and without NAFLD. (**C**) CVH metrics in participants with and without advanced liver fibrosis. (**D**) CVH metrics in participants with and without cirrhosis. CVH, cardiovascular health; NAFLD, non-alcoholic fatty liver disease.

Table 1 shows the baseline characteristics of the included participants. Figure 1A shows the distribution of CVH metrics in the included participants. Among the different categories of CVH metrics, there were significant differences in almost all characteristics except platelet count (*p* = 0.057). Older individuals, men, and singles were more likely to have lower CVH metrics. Poor education levels and CVD were more prevalent among participants with lower CVH metrics. These participants also preferred to have higher levels of platelet count, Hs-CRP, AST, and ALT, while they had lower levels of HDL, eGFR, total bilirubin, and albumin. More importantly, we observed that participants with poor CVH metrics were prone to have higher NAFLD rates (poor score: 51.45%, intermediate score: 26.62%, ideal score: 7.44%, *p* < 0.001), advanced liver fibrosis (poor score: 10.76%, intermediate score: 3.19%, ideal score: 0.60%, *p* < 0.001), and cirrhosis rates (poor score: 4.96%, intermediate score: 1.16%, ideal score: 0.60%, *p* < 0.001). CAP and LSM also decreased with the increase in CVH metrics. Furthermore, participants with NAFLD, advanced liver fibrosis, and cirrhosis tended to have lower CVH metrics (Figure 1B–D).

### 3.2. Association between CAP, LSM, and CVH Metrics

In the unadjusted model (model 1), we found an inverse relationship between CVH metrics and CAP (β = −11.703, 95% confidence interval, CI: (−12.590, −10.816), *p* < 0.001). The inverse association between CVH metrics and CAP remained stable in model 2. In the fully adjusted model (model 3), per 1 unit of increased CVH metrics was associated with 8.565 lower units of CAP (β = −8.565, 95% CI: (−9.601, −7.529), *p* < 0.001) (Table 2). For LSM, there was also a significant inverse relationship between liver stiffness and CVH metrics in all three models (model 1: β = −0.385, 95% CI: (−0.471, −0.300); model 2: β = −0.274, 95% CI: (−0.381, −0.168), *p* < 0.001). The fully adjusted model (model 3) revealed that per 1 unit of increased CVH metrics was associated with 0.274 lower units of LSM (β = −0.274, 95% confidence interval, CI: (−0.381, −0.168), *p* < 0.001) (Table 2).

We also analyzed the CVH metrics as a categorical variable. In model 1, compared to the group with the ideal score, the β with 95% CI of the group with the poor score for CAP and LSM was 72.241 (65.402–79.080) and 2.176 (1.673–2.680), respectively. After adjusting for all covariates, participants with poor scores had increases in CAP and LSM of 45.846 units and 1.593 units, respectively, compared to the participants with ideal scores (95% CI for CAP: 38.444–53.248; 95% CI for LSM: 1.018–2.168) (Table 2).

Figures S1 and S2 show the association between CAP, LSM, and each component of the CVH metrics. Compared to ideal categories, CAP was higher in the participants with poor BMI (β = 56.70, 95% CI: 51.36–62.05), poor blood pressure (β = 9.67, 95% CI: 5.15–14.18), poor total cholesterol (β = 7.11, 95% CI: 1.08–13.14), and poor HbA1c (β = 28.97, 95% CI: 23.06–34.88). There were no significant differences in CAP between poor diet quality, poor physical activity, and poor smoking status and the corresponding ideal categories. Similar results were observed in the association between LSM and each component of the CVH metrics. However, LSM was higher in participants with poor physical activity (β = 0.71, 95% CI: 0.03–1.39) and lower in participants with poor total cholesterol (β = −1.02, 95% CI: (−1.67, −0.36), *p* < 0.001).

### 3.3. Association between NAFLD, Advanced Liver Fibrosis, Cirrhosis and CVH Metrics

We also conducted logistic regression to assess the relationship between NAFLD, advanced liver fibrosis, cirrhosis, and CVH metrics. We observed that CVH metrics were inversely associated with a higher risk of NAFLD, advanced liver fibrosis, and cirrhosis (for NAFLD, odds ratios (OR) = 0.696, 95% CI: 0.668–0.724; for advanced liver fibrosis, OR = 0.700, 95% CI: 0.650–0.751; for cirrhosis: OR = 0.741, 95% CI: 0.669–0.818). This association remained significant after adjusting for age, sex, and race (model 2). Model 3, a fully adjusted model, revealed that an increase in CVH metrics per 1 unit was associated with a 26.2%, 23.9%, and 20.6% decreased risk of NAFLD (OR = 0.738, 95% CI: 0.702–0.776), advanced liver fibrosis (OR = 0.761, 95% CI: 0.697–0.829), and cirrhosis (OR = 0.794, 95% CI: 0.699–0.899), respectively (Table 3).

When the CVH metrics were treated as a categorical variable, participants with poor scores had a higher risk of NAFLD, advanced liver fibrosis, and cirrhosis. Univariate analysis showed that the group with poor scores had a significantly higher OR for NAFLD, advanced liver fibrosis, and cirrhosis than the group with ideal scores. Similar results were also observed in model 2. After adjusting for all covariates, compared to the ideal CVH group, the risk of NAFLD (OR = 7.140, 95% CI: 4.550–11.633), advanced liver fibrosis (OR = 10.687, 95% CI: 3.146–67.225), and cirrhosis (OR = 6.012, 95% CI: 1.609–39.902) were 7, 10, and 6 times higher in the poor CVH group (Table 3).

**Figure 2:**
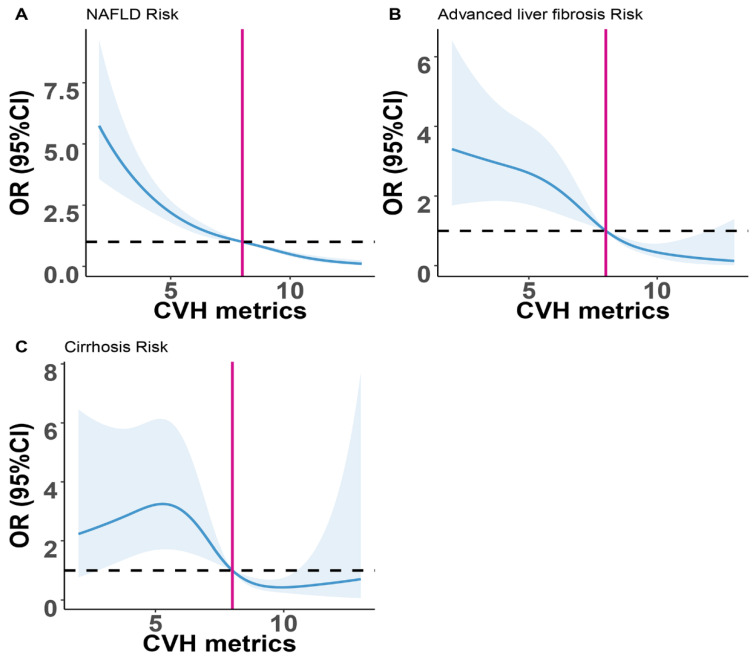
Restricted cubic spline plots of the association of CVH metrics with (**A**) NAFLD, (**B**) advanced liver fibrosis, and (**C**) cirrhosis. The results were adjusted for all covariates. CVH, cardiovascular health; NAFLD, non-alcoholic fatty liver disease.

As shown in Figure 2, the restricted cubic spline was performed to visualize the inverse association between NAFLD, advanced liver fibrosis, cirrhosis, and CVH metrics. In the curve, we found a key point for CVH metrics of 8. The risk of NAFLD, advanced liver fibrosis and cirrhosis was flat if CVH metrics were 8. The finding indicated that we needed to pay more attention to the participants with CVH metrics < 8.

### 3.4. Subgroup Analysis

**Figure 3:**
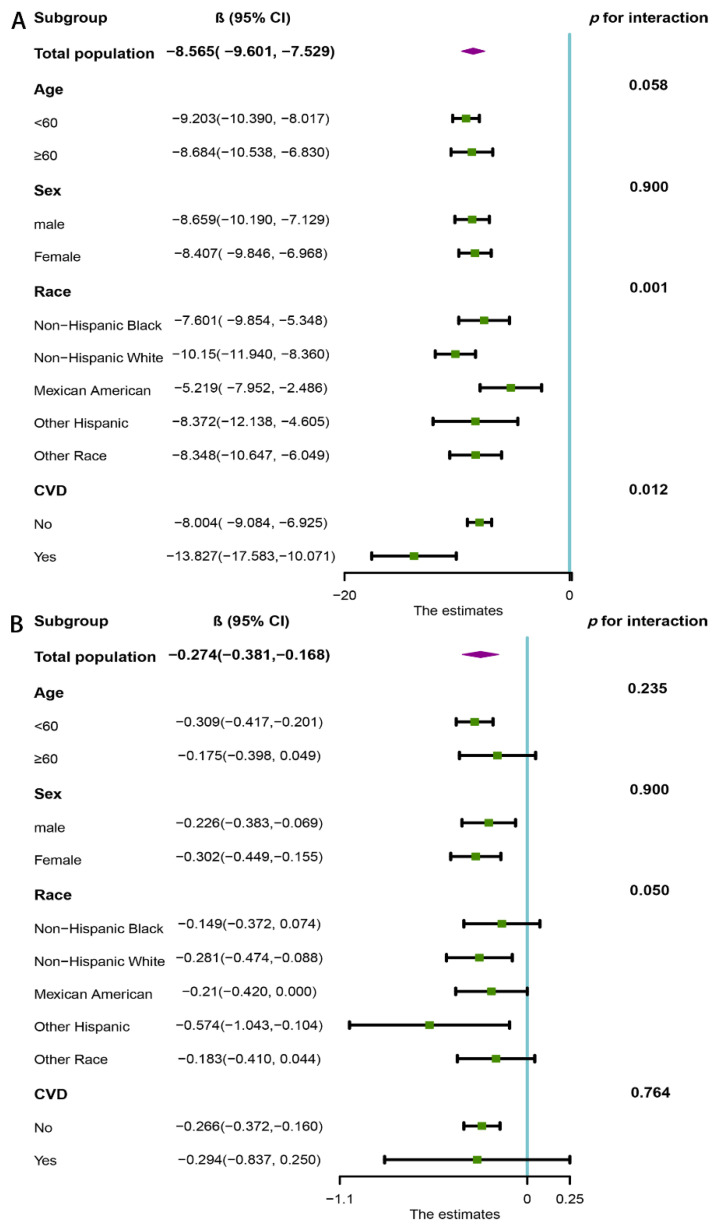
Subgroup analysis of the association of CVH metrics with (**A**) CAP and (**B**) LSM. The results were adjusted for all covariates except for the corresponding stratification variable. CVH, cardiovascular health; CAP, controlled attenuation parameter; LSM, liver stiffness measure.

Subgroup analysis was performed to assess the robustness of the association between CAP, LSM, and CVH metrics. The CVH metrics were treated as a continuous variable. We observed that the inverse association between CAP, LSM, and CVH metrics was stable in the different population settings. Higher CVH metrics were associated with a decrease in CAP in all the stratified subgroups (Figure 3A). Furthermore, the results revealed that the relationship between CAP and CVH metrics was more pronounced in CVD patients (*p* for interaction = 0.012) and non-Hispanic whites (*p* for interaction = 0.001). There was also a significant inverse association between CVH metrics and LSM in most subgroups, whereas this association did not maintain statistical significance in participants aged over 60 years (β = −0.175, 95% CI: −0.398–0.049), non-Hispanic blacks (β = −0.149, 95% CI: −0.372–0.074), Mexican Americans (β = −0.210, 95% CI: −0.420–0.000), other races (β = −0.183, 95% CI: −0.410–0.044), and CVD patients (β = −0.294, 95% CI: −0.837–0.250) (Figure 3B).

## 4. Discussion

This cross-sectional analysis from the large, nationally representative, population-based database demonstrated that CVH metrics were inversely associated with the reduced severity of hepatic steatosis and liver fibrosis. The group with poor CVH was associated with a sevenfold, tenfold, and sixfold increased risk of NAFLD, advanced liver fibrosis, and cirrhosis than the group with ideal CVH. Furthermore, we observed a more pronounced inverse relationship between CAP and CVH metrics in CVD patients and non-Hispanic whites.

To our knowledge, our study was the first to explore the association between CVH metrics and the severity of hepatic steatosis and liver fibrosis detected by FibroScan. Our study further highlighted the link between the liver and CVH and supported previous studies that demonstrated an inverse association between NAFLD and ideal cardiovascular health [4,5,21]. Compared to previous studies investigating the relationship between CVH metrics and NAFLD, a wide range of covariates related to liver function were included in our study, which increased the reliability of our results. Furthermore, we used the ideal CVH metrics as a reference group to further draw the attention of those with poor lifestyles. More importantly, our results suggested that we should pay more attention to CVH metrics in the general population, not only to reduce the risk of CVD but also to reduce the severity of hepatic steatosis and liver fibrosis, as well as the risk of advanced liver fibrosis and cirrhosis.

Lifestyle changes remain the basis of clinical treatment for NAFLD. Emerging studies have explored the role of some components of the CVH metrics on the prevalence of NAFLD and liver fibrosis. Natalia I. Heredia and his colleague reported that diet quality and physical activity were inversely associated with decreased risk of NAFLD, and physical activity might be crucial even for those with advanced liver disease [22]. Similar results were also reported by Eduardo Vilar-Gomez et al. [13]. Notably, a high-quality diet assessed by the HEI score emphasizes the importance of increasing the intake of fruits, vegetables, and a variety of fiber-rich whole grains and seafood, while limiting sodium intake and sugary beverages. Diet may influence liver fat deposition by regulating visceral fat content. Several food components that contain high amounts of water and fiber, such as fruits and vegetables, may improve weight control and increase the production of beneficial short-chain fatty acids (SCFAs) to suppress inflammation, thereby reducing the risk of NAFLD [23]. Physical activity sensitizes skeletal muscle and the liver to insulin response, accelerates glucolipid metabolism in the body, reduces the expression of inflammatory factors, and optimizes the structure of the intestinal flora, thereby decreasing the burden of NAFLD [24,25,26]. Cigarette smoking has also been shown to be strongly associated with NAFLD and may worsen NAFLD by enhancing pro-inflammatory cytokines and oxidative stress [27]. Some studies have reported a positive association between smoking and the severity of liver fibrosis in patients with NAFLD [28,29]. However, we only observed an inverse association between physical activity and LSM among the above three lifestyle factors. This result may be due to the loss of information on categorical variables and the adjustment of other covariates. Although poor physical activity and diet showed a little or no worse effect on hepatic steatosis and liver fibrosis, they had a cumulative effect and were associated with liver health. Furthermore, we noted that former smoking appeared to be more strongly associated with hepatic steatosis and liver fibrosis than current smoking, which is consistent with previous studies [30,31]. Since hepatic steatosis and fibrosis are chronic processes, the extent of liver lesions may be more pronounced in former smokers. It is well known that obesity is also an important element of NAFLD that cannot be ignored. The majority of NAFLD patients are obese, as defined by BMI. BMI was an independent predictor of NAFLD and advanced fibrosis [32]. Recently, an observational study in Taiwan demonstrated that BMI was significantly associated with NASH and the severity of liver fibrosis among NAFLD patients [33]. Our study reported similar results.

In addition to the above four health behaviors, three health factors were also closely associated with the development of NAFLD. Stefano Ciardullo et al. revealed that the prevalence of both liver steatosis and fibrosis is high in patients with type 2 diabetes using the NHANES database [32]. A study using the Duke NAFLD clinical database reported that higher mean HbA1c was associated with higher grades of hepatic steatosis and hepatocyte ballooning. Each 1% increase in mean HbA1c was associated with a 15% increase in the odds of increased liver fibrosis grading [34]. Several studies have similarly confirmed that the prevalence and severity of hypertension were positively associated with the presence and progression of NAFLD [35,36,37,38]. Unlike other health factors, we observed that total cholesterol was positively associated with CAP and negatively associated with LSM. Previous studies have demonstrated that high cholesterol induces adverse effects in liver disorders, such as NAFLD [39]. However, as the synthetic function of the liver decreases due to the formation of fibrosis, cholesterol also decreases. Several studies reported that total cholesterol was inversely associated with NASH and liver fibrosis [40,41]. In summary, NAFLD could be considered a hepatic manifestation of a metabolic syndrome that includes hypertension, hyperglycemia, central obesity, and dyslipidemia [42,43]. Therefore, better control of blood pressure, glucose, lipids, and body weight plays a vital role in reducing the burden of NAFLD and liver fibrosis. However, the above studies mainly focused on individual lifestyle factors associated with the development of NAFLD. Most people are probably more interested in whether and to what extent the combination of the above lifestyle factors affects the severity of hepatic steatosis and liver fibrosis. In addition, few participants can adhere to lifestyle modifications on all sides. Our results revealed that adherence to ideal CVH metrics could significantly reduce the severity of liver steatosis and liver fibrosis, providing a more precise direction for preventing NAFLD and cirrhosis.

Subgroup analysis revealed that the inverse associations between CAP, LSM, and CVH metrics were similar across different ages, sex, race, and CVD status, although not statistically significant in some subgroups. However, we found that the inverse association between CAP and CVH metrics was more pronounced in CVD patients. Both NAFLD and CVD are manifestations of end-organ damage in metabolic syndrome. They are interconnected through multiple pathophysiological mechanisms, such as systemic inflammation, insulin resistance, genetic risk variants, lipid dysregulation, and dysbiosis of the gut microbial ecology [44,45,46]. CVD is the predominant cause of death among patients with NAFLD [47,48]. Patients with NAFLD are also at higher risk of CVD [49]. Therefore, CVD patients could benefit more from ideal CVH. In addition, the results of the subgroup analysis suggest that adherence to ideal CVH metrics is more critical for non-Hispanic whites than for other races. We need to focus more on the CVH metrics for non-Hispanic whites to reduce the severity of hepatic steatosis.

Despite the crucial finding in our results, there are some inherent limitations. First, the causality cannot be concluded from a cross-sectional study. Second, liver biopsy is still the “gold standard” for accurately detecting liver fibrosis. However, the invasive and expensive method cannot be generalized to the public, so liver biopsy data were unavailable in NHANES. The association between CVH metrics and liver biopsy should be explored in future studies. Third, all participants were American, which prevents us from arbitrarily generalizing the findings to other ethnic groups. Finally, cigarette smoking, physical activity, and diet data were obtained using standardized questionnaires, which can lead to recall bias.

## 5. Conclusions

In conclusion, adherence to ideal CVH metrics was associated with a lower risk of hepatic steatosis and liver fibrosis. The ideal CVH metrics also could be a reliable tool to help the general population to reduce the burden of NAFLD and liver fibrosis. Our results could further draw public attention to maintaining a healthy lifestyle.

## Figures and Tables

**Table 1:** Baseline characteristic according to the CVH metrics categories.

Variables	Total (*n* = 2679)	Poor (Score 0–7) (*n* = 1310)	Intermediate (Score 8–10) (*n* = 1033)	Ideal (Score 11–14) (*n* = 3,36)	*p*-Value
Age (years)	50.02 (17.11)	56.62 (14.93)	46.31 (16.67)	35.70 (13.90)	<0.001
Sex, male, *n*, (%)	1348 (50.32%)	715 (54.58%)	517 (50.05%)	116 (34.52%)	<0.001
MS, *n*, (%)					<0.001
Married/living with partner	1634 (60.99%)	806 (61.53%)	643 (62.25%)	185 (55.06%)	
Widowed/divorced/separated	533.00 (19.90%)	334 (25.50%)	170 (16.46%)	29 (8.63%)	
Never married	512.00 (19.11%)	170 (12.98%)	220 (21.30%)	122 (36.31%)	
Education level (%)					<0.001
High school degree/equivalency or less	1026 (38.30%)	554 (42.29%)	375 (36.30%)	97 (28.87%)	
Some college or associates degree	887 (33.11%)	456 (34.81%)	323 (31.27%)	108 (32.14%)	
College graduate or above	766 (28.59%)	300 (22.90%)	335 (32.43%)	131 (38.99%)	
Race, (%)					<0.001
Mexican American	333 (12.43%)	152 (11.60%)	134 (12.97%)	47 (13.99%)	
Non-Hispanic white	970 (36.21%)	493 (37.63%)	371 (35.91%)	106 (31.55%)	
Non-Hispanic black	603 (22.51%)	331 (25.27%)	208 (20.14%)	64 (19.05%)	
Other Hispanic	241 (9.00%)	115 (8.78%)	89 (8.62%)	37 (11.01%)	
Other races	532 (19.86%)	219 (16.72%)	231 (22.36%)	82 (24.40%)	
CVD, (%)	246 (9.18%)	180 (13.74%)	63 (6.10%)	3 (0.89%)	<0.001
Platelet count, 10^9/L	241.98 (62.50)	245.44 (67.35)	239.99 (58.62)	234.65 (52.95)	0.057
Hs-CRP, mg/L	3.84 (6.96)	4.84 (8.16)	3.21 (5.96)	1.88 (2.85)	<0.001
AST, U/L	21.49 (11.09)	21.90 (10.43)	21.23 (12.04)	20.65 (10.52)	0.012
ALT, U/L	21.97 (14.86)	24.00 (16.21)	20.89 (13.71)	17.37 (10.94)	<0.001
HDL, mmol/L	1.38 (0.39)	1.31 (0.38)	1.41 (0.39)	1.56 (0.37)	<0.001
eGFR, mL/min/1.73 m^2^	93.38 (22.61)	86.95 (22.26)	96.97 (21.55)	107.41 (17.89)	<0.001
Total bilirubin, mg/dL	7.97 (4.62)	7.61 (4.34)	8.17 (4.60)	8.76 (5.50)	<0.001
Albumin, g/L	40.79 (3.18)	40.23 (3.25)	41.15 (3.06)	41.85 (2.87)	<0.001
CAP, dB/m	263.60 (62.31)	286.68 (60.71)	250.32 (55.69)	214.44 (45.01)	<0.001
LSM, kPa	5.90 (5.45)	6.76 (6.82)	5.23 (3.78)	4.63 (2.39)	<0.001
NAFLD, (%)	974 (36.36%)	674 (51.45%)	275 (26.62%)	25 (7.44%)	<0.001
Advanced liver fibrosis, (%)	176 (6.57%)	141 (10.76%)	33 (3.19%)	2 (0.60%)	<0.001
Cirrhosis, (%)	79 (2.95%)	65 (4.96%)	12 (1.16%)	2 (0.60%)	<0.001

Values are given as mean (standard deviations) or numbers (percentages). AST, aminotransferase; ALT, alanine aminotransferase; CVD, cardiovascular disease; CVH, cardiovascular health; eGFR, estimated glomerular filtration rate; HDL, high-density lipoprotein; Hs-CRP, high-sensitivity C-reactive protein; MS, marital status; CAP, controlled attenuation parameter; LSM, liver stiffness measure; NAFLD, non-alcoholic fatty liver disease.

**Table 2:** Linear regression model between CAP, LSM, and CVH metrics.

	Model 1		Model 2		Model 3	
	β, (95% CI)	*p*	β, (95% CI)	*p*	β, (95% CI)	*p*
CAP						
Continuous	−11.703 (−12.590, −10.816)	<0.001	−12.059 (−13.038, −11.081)	<0.001	−8.565 (−9.601, −7.529)	<0.001
Categorical						
Poor (0–7)	72.241 (65.402, 79.080)	<0.001	69.683 (62.333, 77.033)	<0.001	45.846 (38.444, 53.248)	<0.001
Intermediate (8–10)	35.885 (28.861, 42.908)	<0.001	33.508 (26.448, 40.569)	<0.001	21.105 (14.368, 27.843)	<0.001
Ideal (11–14)	Reference		Reference		Reference	
LSM						
Continuous	−0.385 (−0.471, −0.300)	<0.001	−0.376 (−0.473, −0.279)	<0.001	−0.274 (−0.381, −0.168)	<0.001
Categorical						
Poor (0–7)	2.176 (1.673, 2.680)	<0.001	2.151 (1.638, 2.664)	<0.001	1.593 (1.018, 2.168)	<0.001
Intermediate (8–10)	0.487 (−0.043, 1.017)	0.072	0.314 (−0.225, 0.854)	0.253	0.220 (−0.331, 0.771)	0.433
Ideal (11–14)	Reference		Reference		Reference	

Data are presented as β, 95% CI (confidence intervals), and *p*-value. Model 1 adjusted for none. Model 2 adjusted for age, sex, race. Model 3 was adjusted for all covariates. CAP, controlled attenuation parameter; LSM, liver stiffness measure.

**Table 3:** Logistic regression model between NAFLD, liver fibrosis, cirrhosis and CVH metrics.

	Model 1		Model 2		Model 3	
	OR (95% CI)	*p*	OR (95% CI)	*p*	OR (95% CI)	*p*
NAFLD						
Continuous	0.696 (0.668, 0.724)	<0.001	0.667 (0.637, 0.698)	<0.001	0.738 (0.702, 0.776)	<0.001
Categorical						
Poor (0–7)	13.183 (8.826, 20.582)	<0.001	14.414 (9.440, 22.925)	<0.001	7.140 (4.550, 11.633)	<0.001
Intermediate (8–10)	4.513 (2.993, 7.099)	<0.001	4.545 (2.990, 7.198)	<0.001	3.098 (1.997, 4.994)	<0.001
Ideal (11–14)	Reference		Reference		Reference	
Advanced liver fibrosis						
Continuous	0.700 (0.650, 0.751)	<0.001	0.707 (0.654, 0.764)	<0.001	0.761 (0.697, 0.829)	<0.001
Categorical						
Poor (0–7)	20.143 (6.381,122.273)	<0.001	15.846 (4.893, 97.230)	<0.001	10.687 (3.146, 67.225)	<0.001
Intermediate (8–10)	5.511 (1.664, 34.099)	0.02	4.769 (1.428, 29.614)	0.033	3.927 (1.141, 24.781)	<0.001
Ideal (11–14)	Reference		Reference		Reference	
Cirrhosis						
Continuous	0.741 (0.669, 0.818)	<0.001	0.732 (0.656, 0.816)	<0.001	0.794 (0.699, 0.899)	<0.001
Categorical						
Poor (0–7)	8.719 (2.715, 53.288)	0.003	8.478 (2.505, 53.012)	0.004	6.012 (1.609, 39.902)	0.022
Intermediate (8–10)	1.963 (0.532, 12.655)	0.379	1.882 (0.503, 12.217)	0.412	1.553 (0.390, 10.466)	0.582
Ideal (11–14)	Reference		Reference		Reference	

Data are presented as odds ratios, 95% CI (confidence intervals), and *p*-value. Model 1 adjusted for none. Model 2 adjusted for age, sex, race. Model 3 was adjusted for all covariates. CVH, cardiovascular health; NAFLD, non-alcoholic fatty liver disease.

## Data Availability

Publicly available datasets were analyzed in this study. These data can be found here: https://wwwn.cdc.gov/nchs/nhanes/ (accessed on 1 October 2022).

## Supplementary Materials

The following supporting information can be downloaded at: https://www.mdpi.com/article/10.3390/nu14245344/s1, Figure S1: Association between CAP and each CVH component; Figure S2: Association between LSM and each CVH component.
