# Supplementary figures and images for: Ideal Cardiovascular Health Metrics Are Associated with Reduced Severity of Hepatic Steatosis and Liver Fibrosis Detected by Transient Elastography
Source: Nutrients. 2022 Dec 16;14(24):5344. doi: 10.3390/nu14245344 (PMC9780817; doi:10.3390/nu14245344)

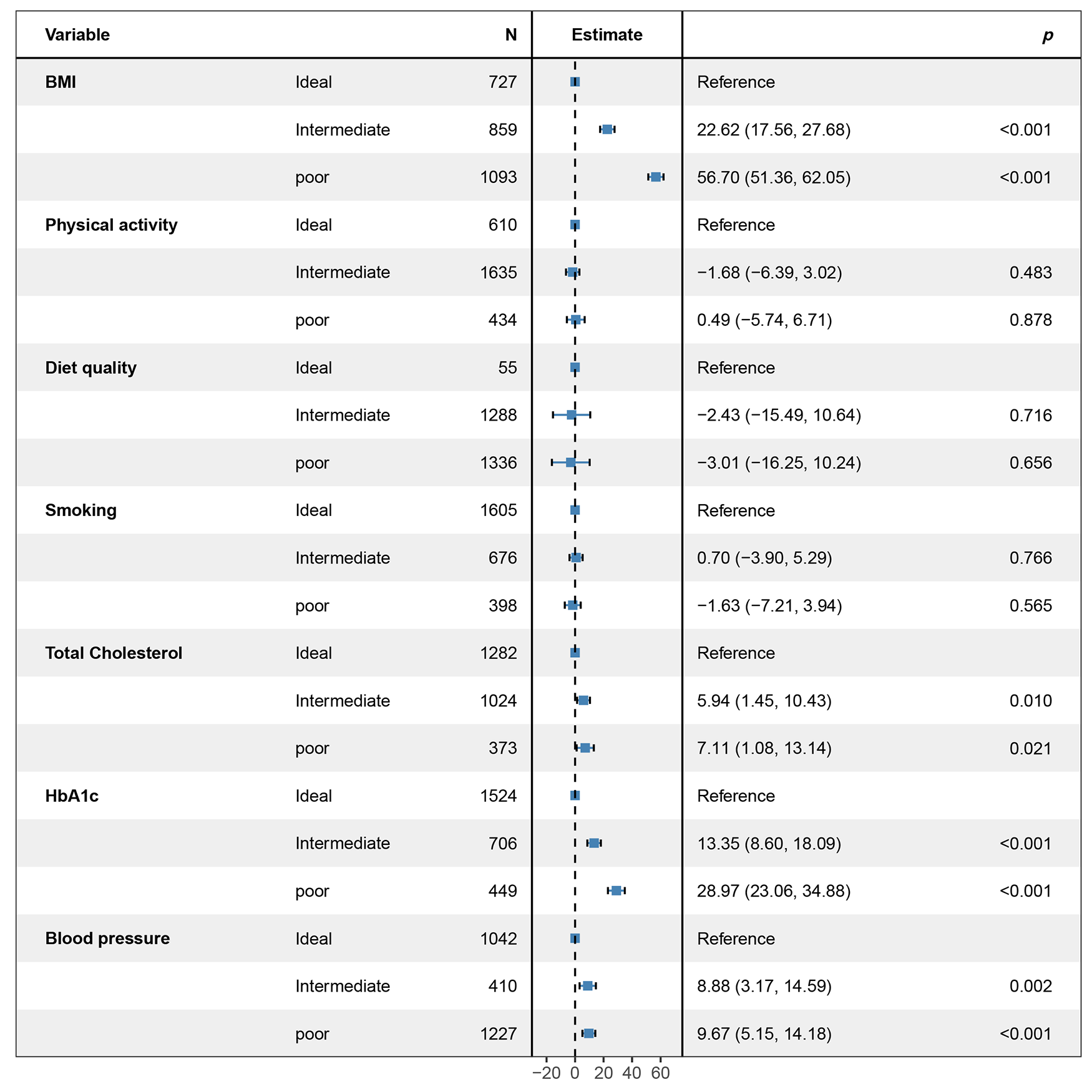

Supplement: Supplementary file 1 [file nutrients-14-05344-s001.zip › Figure S1.tif]

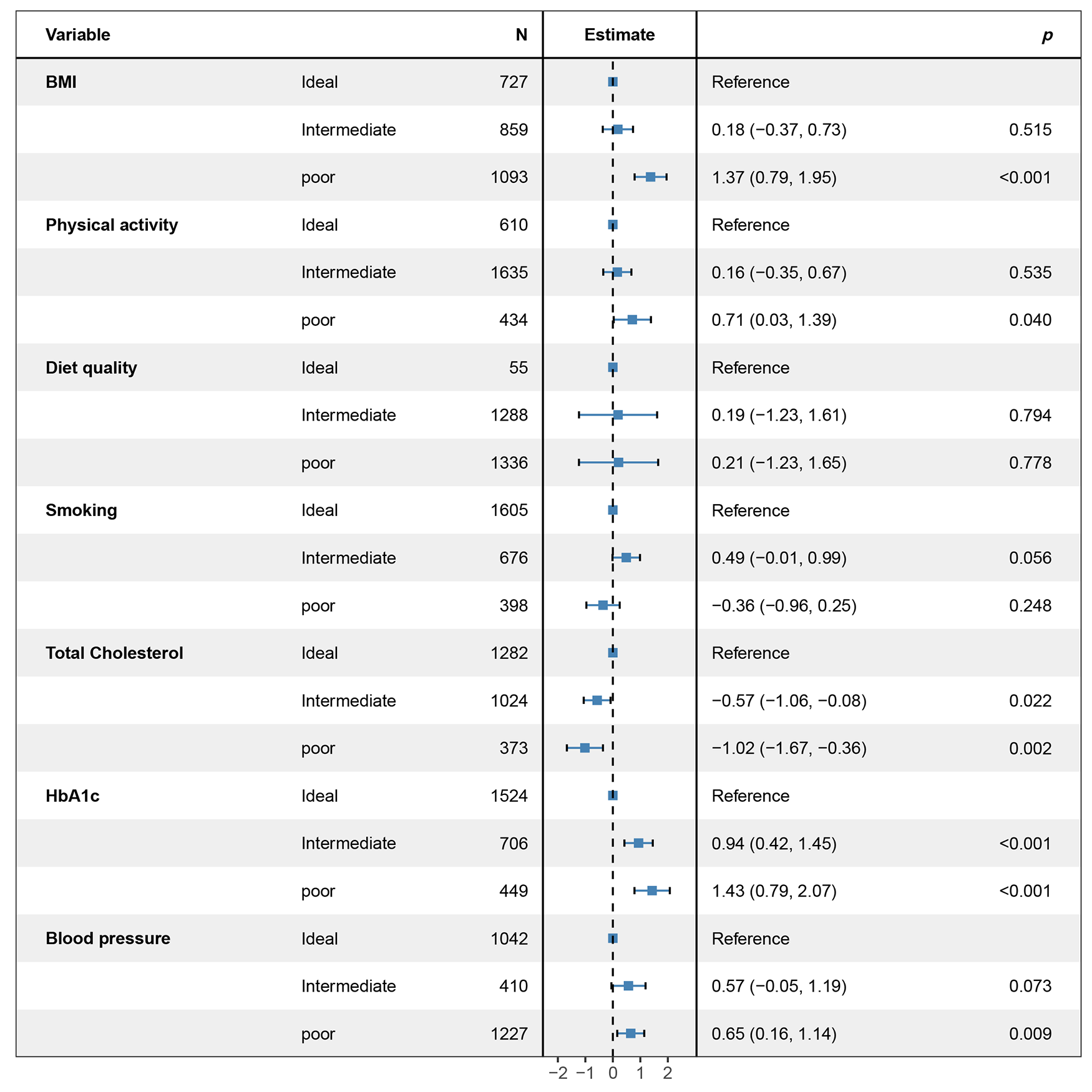

Supplement: Supplementary file 1 [file nutrients-14-05344-s001.zip › Figure S2.tif]
